# Factors associated with neonatal jaundice among neonates admitted at referral hospitals in northeast Ethiopia: a facility-based unmatched case-control study

**DOI:** 10.1186/s12884-024-06352-y

**Published:** 2024-02-21

**Authors:** Tsedale Ayalew, Asressie Molla, Bereket Kefale, Tilahun Dessie Alene, Gebremeskel Kibret Abebe, Habtamu Setegn Ngusie, Alemu Birara Zemariam

**Affiliations:** 1 School of Medicine, College of Medicine and Health Sciences, Wollo University, Dessie, Ethiopia; 2 Department of Epidemiology and Biostatistics, School of Public Health, College of Medicine and Health Sciences, Wollo University, Dessie, Ethiopia; 3 Department of Reproductive and Family Health, School of Public Health, College of Medicine and Health Sciences, Wollo University, Dessie, Ethiopia; 4 Department of Pediatrics and Child Health, School of Medicine, College of Medicine and Health Sciences, Wollo University, Dessie, Ethiopia; 5 Department of Emergency and Critical Care Nursing, School of Nursing, College of Medicine and Health Sciences, Woldia University, Woldia, Ethiopia; 6 Department of Health informatics, School of Public Health, College of Medicine and Health Sciences, Woldia University, Woldia, Ethiopia; 7 Department of Pediatrics and Child Health Nursing, School of Nursing, College of Medicine and Health Sciences, Woldia University, Woldia, Ethiopia

**Keywords:** Bilirubin level, Hyperbilirubinemia, Neonate, Neonatal jaundice, Prematurity, Northeast Ethiopia

## Abstract

**Background:**

Neonatal jaundice is a significant contributor to illness and death in newborns, leading to frequent admissions to neonatal intensive care units. To better understand this issue, a study was conducted to identify the factors contributing to neonatal jaundice among newborns admitted to Dessie and Woldia comprehensive specialized hospitals in northeast Ethiopia.

**Methods:**

The study took place from April 1 to May 30, 2022, using unmatched case-control design. A total of 320 neonates paired with their mothers were involved, including 64 cases and 256 controls. Data were collected through a structured interviewer-administered questionnaire and a review of medical records. The collected data were analyzed using SPSS Version 23, and a multivariate logistic regression model was employed to understand the relationship between independent factors and the occurrence of neonatal jaundice. Statistical significance was determined at a threshold of *P* value less than 0.05.

**Results:**

The study findings revealed that maternal age over 35 years, residing in urban areas [adjusted odds ratio (AOR) = 2.4, 95% confidence interval (CI): 1.23, 4.82], male gender (AOR = 4.3, 95% CI: 1.90, 9.74), prematurity (AOR = 3.9, 95% CI: 1.88, 8.09), and ABO incompatibility (AOR = 2.6, 95% CI: 1.16, 5.96) were significant determinants of neonatal jaundice. Conversely, the study indicated that cesarean birth was associated with a 76% lower likelihood of infant jaundice compared to vaginal delivery (AOR = 0.24, 95% CI: 0.08, 0.72).

**Conclusion:**

To prevent, diagnose, and treat neonatal jaundice effectively, efforts should primarily focus on managing ABO incompatibility and early detection of prematurity. Additionally, special attention should be given to neonates born through vaginal delivery, those with mothers over 35 years old, and those residing in urban areas, as they are at higher risk of developing newborn jaundice. Close monitoring of high-risk mother-infant pairs during the antenatal and postnatal periods, along with early intervention, is crucial for reducing the severity of neonatal jaundice in this study setting.

## Introduction

Jaundice refers to the yellowish discoloration of the skin, sclerae, and mucous membranes caused by the accumulation of bilirubin in the tissues [1, 2]. In older children and adults, a total blood bilirubin level exceeding 1.5 mg/dL is considered hyperbilirubinemia [3]. However, during the postpartum phase of transition, infants experience physiological jaundice, where their blood bilirubin levels surpass 1.5 mg/dL due to normal transitional processes [4, 5]. Jaundice becomes detectable clinically when the total blood bilirubin level reaches 5 mg/dL [3, 6].

If the timing, duration, or pattern of jaundice significantly differs from physiological jaundice, or if the infant shows additional indications of susceptibility to neurotoxicity, the jaundice and its underlying hyperbilirubinemia are considered pathological. The progression of jaundice, measured in milligrams per deciliter (mg/dL), typically starts with the face (5 mg/dL), followed by the mid-abdomen (15 mg/dL), and the soles of the feet (20 mg/dL) [7, 8].

Severe hyperbilirubinemia affects approximately 1.1 million infants worldwide each year, with the majority residing in sub-Saharan Africa and South Asia [9]. Neonatal jaundice ranks as the seventh and eighth leading causes of death in South Asia and sub-Saharan Africa, respectively [10]. In Africa, neonatal jaundice is a significant contributor to neonatal admissions [11]. and Ethiopia is among the top ten countries globally in terms of infant deaths from jaundice [11].

Several risk factors for neonatal jaundice have been identified, including prematurity [12], maternal age [13, 14], low birth weight [15], glucose-6-phosphate dehydrogenase deficiency (G6PD) [16], genetics [17], sex [18], drugs, race [19], altitude, polycythemia [15], maternal diabetes [20], blood extravasation cutaneous bruising [21],, oxytocin induction [13, 16], delayed bowel movement, family history of physiological jaundice [18], breast milk, weight loss [21], blood-group incompatibilities [22], and other hemolytic diseases [23].

Various interventions have been implemented to reduce the morbidity and mortality associated with neonatal jaundice. These interventions include genetic screening to identify enzymatic deficiencies, educating mothers to recognize the early signs of jaundice and seek timely healthcare services, and conducting blood group screening during antenatal care (ANC) with the provision of Anti D for mothers who have Rh negative status during pregnancy and postpartum [24]. Furthermore, guideline development and implementation have been among the strategies employed to enhance perinatal and neonatal health outcomes [25]. To combat this neonatal complication, the Ethiopian government has taken steps to ensure the accessibility of phototherapy treatment in hospitals. Currently, the government of Ethiopia has set a target to reduce the neonatal mortality rate from 28 per 1000 live births to 11 per 1000 live births by 2035, as indicated in the Ministry of Health report [26].

Limited research has been conducted to identify the factors contributing to neonatal jaundice in Ethiopia. One study conducted in Mekelle City, located in northern Ethiopia, reported a prevalence of 37.3% for newborn jaundice [27]. Another case-control study conducted in northern Ethiopia examined intervention strategies for addressing neonatal jaundice [15]. Previous studies assessing the determinants of newborn jaundice [21, 28, 29], often did not utilize statistical regression models to identify predictors. Moreover, there were conflicting findings regarding predictors such as mode of delivery and their association with neonatal jaundice. Therefore, this study aims to address the aforementioned gaps and determine the causes of neonatal jaundice.

## Method

### Study area and period

The study was conducted at Woldia and Dessie Referral Hospitals from April 1 to May 30, 2022. Woldia and Dessie are the capital cities of the North and South parts of Wollo, respectively. Dessie is located approximately 401 km north of Addis Ababa, the central city of Ethiopia, while Woldia is situated about 521 km away. Dessie Referral Hospital serves a catchment population of over 5 million people, including areas in South Wollo, Amhara, Tigray, Afar, and Oromia Regions. The hospital has a large staff of more than 500 individuals working in various departments, including administrative personnel. Specifically, the pediatrics department at Dessie Referral Hospital is staffed with 5 pediatricians, 20 general practitioners, and over 40 nurses. The department is equipped with four rooms, 20 beds, and three phototherapy machines.

On the other hand, Woldia Referral Hospital is a public healthcare facility that provides services to approximately 3 million people. The hospital has a staff of over 400 individuals in different departments, including administrative personnel. Currently, two pediatricians are working at Woldia Referral Hospital. The hospital’s Neonatal Intensive Care Unit (NICU) has 15 beds and four phototherapy machines.

### Study design and population

We carried out a facility-based unmatched case-control study among cases and controls admitted in NICU at Dessie and Woldia referral hospitals. For cases, our source population was jaundiced neonates who came to the NICU of Dessie and Woldia referral hospitals and their mothers. On the other hand, the source population for controls was all neonates seeking care but without jaundice who came to the NICU of Dessie and Woldia comprehensive specialized hospitals to seek any clinical care and their mothers. For instance, a neonate who came to the hospital for asphyxia, sepsis, birth trauma, surgical problems, and so on was considered the reason for the admittance of the control group.

The study population for cases was all neonates who had jaundiced and were admitted to the NICU of Dessie and Woldia referral hospitals within the study period and their mothers. For controls, the study population was all neonates seeking care, but without jaundice admitted to the NICU ward at Dessie and Woldia referral hospitals within the study period and their mothers. The exclusion criteria for the case were neonates with jaundice who were critically ill, whose mothers were not around, and who had critically ill mothers. In line with this, neonates without jaundice whose mothers were not around and who had critically ill mothers were excluded from the control group.

### Sample size determination and sampling procedure

We calculated the sample size using Epi Info version 7 by using predictors, which were identified in previous studies of similar settings to that of our study area or resource-limited settings due to the scarcity of research for identified factors in Ethiopia [30], and finally, the largest was taken. The sample size was calculated for each determinant factor using the following formula:

$$n = (\frac{{r + 1}}{r})\frac{{(\bar p)(1 - \bar p){{({Z_\beta } + {Z_{\alpha /2}})}^2}}}{{{{({p_1} - {p_2})}^2}}}$$

Where n = number of cases, r = ratio of controls to cases, ZB = power, Za/2 = level of statistical significance, *P* = average proportion exposed, and *P* 1- *P* 2 = difference in proportions.

A 95% level of certainty, a power of 80%, and a ratio of case-to-control 1:4 were considered. After calculating for each factor, we took the largest sample size, which was calculated using prolonged labor [30], the percent of the case exposed was 43%, and the percent of control exposed was 24%. Accordingly, the final sample size became 320 (case = 64, control = 256). A total of 320 neonates paired with their mothers were approached in the study.

The two hospitals are among the public hospitals in the area that give NICU service to a larger catchment population and treat neonates having jaundice. We included all neonates who presented to NICU wards with or without neonatal jaundice and their mothers in the study. All neonates who met the inclusion criteria for the study were included in the cases using sequential sampling until we obtained the required sample size. For each case identified during data collection, four controls were included because we used a case-to-control ratio of 1:4. We also used systematic sampling to select controls from neonates who came to NICU to seek any care but didn’t have jaundice, such as asphyxia, sepsis, birth trauma, surgical problems, and so on. We calculated the sampling interval (k) using the estimation of the total neonates to be seen in the study period from previous experience.

On average, there were ten admissions per day at Dessie Referral Hospital and eight admissions daily at Woldia Referral Hospital. Therefore, the total admissions estimated in the study period became 1,008. Then we calculated the sampling interval by dividing it by the sample size (1008/256). We got K = 4. We used the lottery method to select the first neonate and included every 4th neonate who presented to the hospitals at the time of the data collection period. Cases were neonates diagnosed as having jaundice by the pediatrician or general practitioner using a physical examination. Controls were neonates diagnosed as not having jaundice by the pediatrician or general practitioner using the physical examination.

### Variables

The outcome variable was neonatal jaundice. The explanatory variables were maternal socio-demographic variables, maternal obstetric, and clinical variables (including parity, complications during pregnancy, blood group incompatibility, and previous history), labor and delivery-related variables (including prolonged labor, mode of delivery, induction of labor, oxytocin use for labor augmentation) and neonatal variables (including sex of the infant, prematurity, birth weight, birth asphyxia, hypothermia, sepsis, breastfeeding, cephalohematoma, polycythemia).

### Operational definition

#### Neonatal jaundice

It can be operationally defined as the condition characterized by the yellowing of the newborn’s skin and the whites of the eyes, resulting from the accumulation of bilirubin in the bloodstream. Diagnosis is typically made by a medical professional through a physical examination, specifically observing the presence of yellow discoloration. Laboratory investigation is performed to confirm the diagnosis, typically involving the measurement of total serum bilirubin levels. A commonly used threshold for diagnosing neonatal jaundice is a total serum bilirubin level of 5 mg/dL (85 μmol/L) or higher, although specific thresholds may vary depending on the infant’s age, gestational age, and other factors [31–33].

#### Neonatal sepsis

Neonatal sepsis was identified using a set of established clinical features known as the Integrated Management of Neonatal and Childhood Illness (IMNCI) criteria. These criteria encompass a range of indicators that healthcare providers use to evaluate newborns for sepsis. Specifically, the presence of two or more of the following signs is considered: a persistent fever (≥ 37.5 °C) or prolonged hypothermia (≤ 35.5 °C) lasting for over an hour, rapid breathing (≥ 60 breaths per minute), severe chest indrawing, grunting, insufficient feeding, minimal movement unless stimulated, a bulging fontanelle, convulsions, lethargy, or unconsciousness. Additionally, at least two hematological criteria are taken into account, including the total leukocyte count (less than < 4000 or greater than > 12,000 cells/mm3), absolute neutrophil count (less than < 1500 cells/mm3 or greater than > 7500 cells/mm3), platelet count (less than < 150 or greater than > 450 cells/mm3), and random blood sugar (less than < 40 mg/dl or greater than > 125 mg/dl). By evaluating these clinical and hematological factors, healthcare professionals can make a diagnosis of neonatal sepsis [34–36].

#### Polycythemia

It is defined as a condition characterized by an elevated hematocrit level or hemoglobin level in a peripheral venous blood sample. Specifically, polycythemia is diagnosed when the hematocrit level is equal to or greater than 65% or when the hemoglobin level is equal to or greater than 22 g/dL. These thresholds serve as indicators of increased red blood cell mass in the bloodstream. It is important to note that the diagnosis of polycythemia should consider other clinical factors, such as symptoms and medical history, to confirm the presence of the condition and rule out other potential causes [15, 37, 38].

#### Premature rupture of membranes (PROM)

It is an amniotic sac ruptured before the onset of labor. Preterm premature rupture of membranes (PPROM) is the term used when PROM occurs before 37 weeks of pregnancy [39].

#### Preeclampsia

It is a pregnancy-specific hypertensive disorder characterized by the onset of high blood pressure after 20 weeks of gestation in a previously normotensive woman. It is typically accompanied by proteinuria (excess protein in urine), end-organ dysfunction (such as renal, hepatic, neurological, or hematological abnormalities), and/or placental dysfunction (including fetal growth restriction or abnormal umbilical artery Doppler velocimetry) [40].

#### High blood pressure

It is often known as hypertension, and it is a frequent illness where the blood’s long-term strain against your artery walls is so great that it may eventually result in health issues like heart disease [41]. Hypertension in pregnancy is characterized as two separate occurrences of blood pressure > 140/90 mm Hg [42].

#### Antepartum hemorrhage (APH)

It refers to the occurrence of bleeding from or into the vaginal tract that begins at or before the 24th week of pregnancy and continues until the delivery of the baby. This condition involves the presence of vaginal bleeding during the period leading up to childbirth. The bleeding can be either evident or concealed and detectable only through medical tests or imaging. It is crucial to seek medical attention and evaluation for APH, as it can have various underlying causes and may pose risks to the health and well-being of both the mother and the developing fetus [43].

#### Intrauterine growth restriction (IUGR)

When a fetus (a baby in the womb) does not grow as expected, this condition is known as intrauterine growth restriction (IUGR) [44].

#### Hypothermia

When your body loses heat more quickly than it can produce it, a medical emergency known as hypothermia occurs, resulting in a dangerously low body temperature. A neonate of a newborn was considered hypothermic if the axillary temperature was below 36.5 °C [45–47].

#### ABO incompatibility

When a mother’s blood type is O, and her child has an A, B, or AB blood type, this condition is known as ABO incompatibility [48]. In this investigation, the presence of at least two of the following conditions was required for ABO incompatibility: (1) different degrees of anemia, (2) red blood cells with nuclei in circulation, (3) spherocytosis, or (4) polychromasia on a peripheral blood smear [49].

#### Critically ill neonates

In this study, critically ill neonates are defined as newborn infants who present with severe clinical manifestations or laboratory abnormalities related to jaundice, requiring immediate medical attention and intensive medical interventions [50, 51].

### Data collection instruments and quality control

The data for this study was collected by a team of four BSc nurses through interviews with the mothers of the neonates and by reviewing the corresponding medical records. To gather the required information, a structured questionnaire and checklist were used. These tools were developed by the principal investigator and included predictors identified in previous studies on this topic [30].

The study encompassed various aspects, including socio-demographic characteristics, obstetric characteristics of the mother, and neonatal characteristics. Two medical doctors were assigned as supervisors to oversee the research. To maintain consistency, the questionnaire was initially translated from English to Amharic for data collection purposes and then back-translated to English. This process underwent a pre-test where 5% of the total sample size was tested at Boru-Meda Hospital. Based on the feedback received during the pre-testing phase, relevant changes and modifications were made to the questionnaire. Data collectors underwent a one-day training session covering the entire data collection process. Throughout the data collection period, the supervisors diligently reviewed the completed questionnaires to ensure the accuracy, completeness, and clarity of the data.

### Data analysis

We entered the collected data into EpiData version 4.6 and then exported it to SPSS 23 for analysis. Descriptive analysis such as frequencies, cross tab, and mean were done. A binary logistic regression model was then used in a bivariate study to determine the relationship between each determinant and the outcome variable. The multivariable analysis model then includes variables from the bivariate analysis with a *P*-value of less than or equal to 0.25. The assumptions of logistic regression were fulfilled, the constant for variables in the equation was significant (*P* < 0.05), model prediction of variables improved from 80 to 84.7%, and we checked the model fitness using the Hosmer Lemeshow test and it was not significant (*P* = 0.716). In the multivariable binary logistic regression, a *P*-value of less than 0.05 was utilized as a cut point to assess the level of statistical significance. The adjusted odds ratio (AOR) and 95% confidence interval (CI) were estimated to see the strength of the relationship.

## Results

### Socio-demographic characteristics

About 320 (case = 64, control = 256) mother and neonate pairs participated in the study, making the response rate 100%. The age of mothers included in this study ranged from 18 to 40 years. The mean age of mothers in the cases and controls were 27.3 (SD ± 3.86) and 26.7 (SD ± 5.39) years, respectively. Of the respondents, 63(98.4%) cases and 246(96.1%) controls were married. Of the cases involved in the study, 43(67.2%) were from urban areas. Among neonates included in the study, 24(37.5%) cases and 163(63.7%) controls were admitted on their first day of birth. Regarding the sex of the neonates, 53(82.8%) cases and 152(59.4%) controls were males (See Table 1).

**Table 1:** Socio-demographic characteristics of study participants in Dessie and Woldia Referral Hospital, northeast Ethiopia, 2022

Variable	Category	Case *n* = 64(%)	Control *n* = 256(%)
Mother’s age	< 25years	14(21.9%)	83(32.4%)
	25–35 years	36(56.2%)	165(64.5%)
	> 35 years	14(21.9%)	8(3.1%)
Place of residence	Urban	43(67.2%)	124(48.4%)
	Rural	21(32.8%)	132(51.6%)
Marital status	Married	63(98.4%)	246(96.1%)
	Other*	1(1.6%)	10(3.9%)
Level of education	Cannot read and write	9(14.1%)	34(13.3%)
	Read and write	5(7.8%)	29(11.3%)
	Primary education	18(28.1%)	77(30.1%)
	Secondary education	17(26.6%)	80(31.2%)
	College and above	15(23.4%)	36(14.1%)
Occupation	Housewife	44(68.8%)	184(71.9%)
	Government worker	10(15.6%)	27(10.5%)
	Merchant	6(9.4%)	22(8.6%)
	Other**	4(6.2%)	23(9%)
Age of neonate	< 2 days	24(37.5%)	163(63.7%)
	2–7 days	36(56.2%)	66(25.8%)
	> 7days	4(6.3%)	27(10.5%)
Sex of neonate	Male	53(82.8%)	152(59.4%)
	Female	11(17.2%)	104(40.6%)

Note: * =Single, divorced, and widow **student, farmer, daily laborer, and private employee

### Obstetric and clinical characteristics of the mother

Among mothers included in the study, 43(67.2%) cases and 136(53.1%) controls were multi-parous. Eight (12.5%) mothers participated in the study as cases had a previous history of a child with neonatal jaundice. All cases and 254(99.2%) controls had ANC follow-up during pregnancy. Among participant mothers, 33(51.6%) cases and 76(29.7%) controls had a complication during pregnancy. The commonest complication was PROM, 20(60.6%) from cases and 26(34.2%) from controls. All participants gave birth at a health facility. All cases and 242 (94.5%) controls had labor lasting less than 24 h. Regarding mode of delivery, 9(14.1%) of cases and 23(9%) of controls gave birth via instrumental delivery. The most common blood group and Rh of mothers were O-positive with a total of 113 respondents, which include 31(48.4%) cases and 82(32%) controls (See Table 2).

**Table 2:** Obstetric and clinical characteristics of the mothers in Dessie and Woldia Referral Hospitals, northeast Ethiopia, 2022

Variable	Category	Case *n* = 64(%)	Control *n* = 256(%)
Parity	Primiparous	21(32.8%)	120(46.9%)
	Multiparous	43(67.2%)	136(53.1%)
Previous history of a child with neonatal jaundice	Yes	8(12.5%)	1(0.4%)
	No	56(87.5%)	255(99.6%)
Any complications during pregnancy?	Yes	33(51.6%)	76(29.7%)
	No	31(48.4%)	180(70.3%)
Type of complication	PROM	20(60.6%)	26(34.2%)
	Preeclampsia/hypertension	9(27.2%)	26(34.2%)
	Anemia	2(6.1%)	4(5.3%)
	Other*	2(6.1%)	20(26.3%)
Rh status of the mother	Positive	54(84.4%)	237(92.6%)
	Negative	10(15.6%)	19(7.4%)
Onset of labor	Spontaneous	60(93.7%)	218(85.2%)
	Induced	3(4.7%)	20(7.8%)
	No labor	1(1.6%)	18(7%)
Oxytocin use during labor	Yes	18(28.1%)	47(18.4%)
	No	46(71.9%)	209(81.6%)
Presentation	Normal	63(98.4%)	241(94.1%)
	Malpresentation	1(1.6%)	15(5.9%)
Mode of delivery	Vaginal delivery	50(78.1%)	185(72.3%)
	C/S	5(7.8%)	48(18.7%)
	Instrumental delivery	9(14.1%)	23(9%)

Note: *=DM, APH, multiple pregnancies, IUGR, oligohydramnios, chorioamnionitis

### Neonatal characteristics

Among neonates included in the study, 33(51.6%) cases and 72(28.1%) controls had low birth weights (< 2500 g). Concerning gestational age, 30(46.9%) of cases and 57(22.3%) of controls were preterm neonates (gestational age < 37 weeks). From neonates who had birth trauma, cephalohematoma was the most common one, with 10(76.9%) cases and 10(47.6%) controls. The majority of the neonates had hypothermia, and from those with hypothermia, 50(78.1%) were cases, and 221(86.3%) were among the control.

Among neonates who had jaundice, the range of their serum total bilirubin level was 16.4 to 40.6 mg/dl, and the mean bilirubin level was 21.47 mg/dl (+ 4.67). The majority, 42(65.6%) of cases range a bilirubin level of 16.4–19.9. About 19 (29.7%) had a bilirubin level between 20 and 24.9. On the left, 3 (4.7%) of the cases had a bilirubin level above 25. Among those who had jaundice, 38 (59.4%) cases were pathological neonatal jaundice, and the left 26 (40.6%) were found to be physiological jaundiced. Additionally, only 10 (15.6%) infants were treated with double exchange transfusion. The left 54 (84.4%) were treated with phototherapy due to the serum bilirubin level reaching the phototherapy range based on the butane curve [32, 52].

Among 256 controls, 64(25.0%) were admitted with asphyxia and sepsis, 147 (57.4%) neonates were admitted with sepsis, 13(5.1%) were admitted with birth trauma like cephalohematoma, subgaleal hemorrhage, and other. Additionally, 10 (3.9%) were admitted with anemia, and 22 (8.6%) were admitted with surgical problems. The common blood group was A with Rh positive found among a total of 104 respondents, which included 15(23.4%) cases and 89(34.8%) controls (See Table 3).

**Table 3:** Neonatal Characteristics of study participants in Dessie and Woldia Comprehensive Specialized Hospital, northeast Ethiopia, 2022

Variable	Category	Case *n* = 64(%)	Control *n* = 256(%)
Birth weight	< 2500 gm	33(51.6%)	72(28.1%)
	≥ 2500 gm	31(48.4%)	184(71.9%)
Feeding	Exclusive breastfeeding	52(81.2%)	185(72.3%)
	Formula Milk	3(4.7%)	12(4.7%)
	Nothing per oral	9(14.1%)	59(23%)
Gestational age	< 37 weeks	30(46.9%)	57(22.3%)
	≥ 37 weeks	34(53.1%)	199(77.7%)
Birth asphyxia	Yes	12(18.8%)	64(25%)
	No	52(81.2%)	192(75%)
Is there ABO incompatibility?	No	47(73.4%)	210(82%)
	Yes	17(26.6%)	46(18%)
Birth trauma	Yes	13(20.3%)	21(8.2%)
	No	51(79.7%)	235(91.8%)
Type of birth trauma	Cephalo-hematoma	10(76.9%)	10(47.6%)
	Other*	3(23.1%)	11(52.4%)
Neonatal sepsis	Yes	49(76.6%)	201(78.5%)
	No	15(23.4%)	55(21.5%)
Polycythemia	Yes	1(1.6%)	7(2.7%)
	No	63(98.4%)	249(97.3%)

Note: *=subgaleal hemorrhage, facial edema, and shoulder dislocation

### Determinant factors of neonatal jaundice

During multivariable analysis among the independent variables, residence, sex of the neonate, mother’s age, mode of delivery, gestational age, and presence of ABO incompatibility between the neonate and mother were found to be independent predictors of neonatal jaundice.

Hence, the probability of having a neonate with jaundice is 8.8 times higher for mothers older than 35 years of age compared to those younger than 25 years [AOR = 8.8, 95%CI: (1.99, 38.78)]. Respondents who lived in urban areas were 2.4 times more likely to have neonatal jaundice than those from rural areas [AOR = 2.4, 95%CI: (1.23, 4.82)]. Additionally, male neonates were found to have a 4.3 times higher likelihood of having neonatal jaundice than female neonates [AOR = 4.3, 95%CI: (1.90, 9.74)].

This study showed that cesarean delivery was 76% protective of neonatal jaundice compared with vaginal delivery [AOR = 0.24, 95%CI: (0.08, 0.72)]. Whereas premature neonates (Gestational age less than 37 weeks) had a 3.9 times higher probability of having jaundice than those with gestational age greater than or equal to 37 weeks [AOR = 3.9, 95%CI: (1.88, 8.09)]. Finally, neonates who had ABO incompatibility had 2.6 times more likely to have jaundice than those who had no ABO incompatibility [AOR = 2.6, 95%CI: (1.16, 5.96)] (See Table 4).

**Table 4:** Multivariable logistic regression, neonatal jaundice, Dessie and Woldia Referral Hospitals, northeast Ethiopia, 2022

Variable	Category	Case (*n* = 64)	Control (*n* = 256)	COR (95% CI)	AOR (95% CI)
Age of the mother	< 25 years	14(21.9%)	83(32.4%)	1	1
	25–35 years	36(56.2%)	165(64.5%)	1.3(0.66–2.53)	1.5(0.65–3.56)
	> 35 years	14(21.9%)	8(3.1%)	10.4(3.68–29.26)	8.8(1.99–38.78)*
Place of residence	Urban	43(67.2%)	124(48.4%)	2.2(1.23–3.88)	2.4(1.23–4.82)*
	Rural	21(32.8%)	132(51.6%)	1	1
Sex of neonate	Male	53(82.8%)	152(59.4%)	3.3(1.64–6.61)	4.3(1.90–9.74)**
	Female	11(17.2%)	104(40.6%)	1	1
Parity	Primiparous	21(32.8%)	120(46.9%)	1	1
	Multiparous	43(67.2%)	136(53.1%)	1.8(1.01–3.22)	1.42(0.65–3.10)
Any complications during pregnancy?	Yes	33(51.6%)	76(29.7%)	2.5(1.44–4.4)	1.92(0.94–3.90)
	No	31(48.4%)	180(70.3%)	1	1
Mode of delivery	Vaginal delivery	50(78.1%)	185(72.3%)	1	1
	C/S	5(7.8%)	48(18.7%)	0.4(0.15–1.02)	0.24(0.08–0.72)*
	Instrumental	9(14.1%)	23(9%)	1.4(0.63–3.32)	2.1(0.74–5.71)
Oxytocin use during labor	Yes	18(28.1%)	47(18.4%)	1.7(0.93–3.27)	1.8(0.79–4.10)
	No	46(71.9%)	209(81.6%)	1	1
Gestational age	< 37 weeks	30(46.9%)	57(22.3%)	3.1(1.74–5.46)	3.9(1.88–8.09)**
	≥ 37 weeks	34(53.1%)	199(77.7%)	1	1
Rh status of the mother	Positive	54(84.4%)	237(92.6%)	1	1
	Negative	10(15.6%)	19(7.4%)	2.3(1.02–5.25)	2.2(0.82–5.92)
ABO incompatibility	Yes	17(26.6%)	46(18%)	1.6(0.87–3.13)	2.6(1.16–5.96)*
	No	47(73.4%)	210(82%)	1	1

Note: * =significant at *p* ≤ 0.05, **= significant at *P* < 0.001, COR = Crude Odds Ratio, AOR = Adjusted Odds Ratio

## Discussion

This facility-based, unmatched case-control study was conducted to identify the causes of newborn jaundice. The study’s findings provided valuable insights, revealing that mothers aged 35 years and older had a significantly higher likelihood of giving birth to neonates with jaundice when compared to mothers younger than 25 years. This finding is supported by previous studies conducted in Sweden and Tehran [13, 14]. One potential explanation for this finding is that older mothers, who are 35 years and older, have a higher likelihood of experiencing medical conditions like gestational diabetes and hypertensive disorders. These conditions can affect how the liver functions and how bilirubin is metabolized in the body, leading to an increased risk of neonatal jaundice [53–55]. Additionally, older mothers also have a higher incidence of genetic factors associated with neonatal jaundice, such as genetic disorders and variations in genes related to bilirubin metabolism [55–57]. These combined factors contribute to the heightened probability of neonatal jaundice in neonates born to mothers aged 35 years and older. Therefore, it is important to screen women over 35 years of age before conception and provide enhanced follow-up care during pregnancy, labor, and delivery.

The study also revealed that, after adjusting for other factors, respondents residing in urban areas were twice as likely to develop neonatal jaundice compared to those from rural areas. This difference could be explained by the fact that mothers in urban areas tend to bring their babies to healthcare facilities at an earlier stage when they notice any changes, resulting in a higher diagnosis rate in urban areas compared to rural areas. Conversely, rural mothers often follow traditional practices and stay at home in darker room setups during the postpartum period, which may prevent them from noticing the signs of jaundice. As a result, there may be a higher reporting of jaundice cases in urban areas, leading to a significant effect. Additionally, rural mothers may face challenges in accessing healthcare facilities due to distance, leading them to rely on traditional home remedies instead of seeking medical care. A study conducted in Northern Ethiopia revealed a significant disparity in knowledge levels between urban and rural mothers, bolstering our justification that urban mothers possess a greater understanding of the importance of seeking medical care for their neonates upon observing symptoms related to jaundice. The findings underscore the notion that urban mothers are more informed and equipped to recognize the signs of jaundice in their infants, prompting them to take proactive measures such as seeking medical attention promptly [58].

Based on the implication of this finding, future researchers are encouraged to investigate the underlying factors that contribute to the difference in neonatal jaundice prevalence between urban and rural areas. They should also explore the influence of cultural practices and conduct comparative studies to understand variations across regions. The sex of the neonate was identified as an important predictor of newborn jaundice. The study found that male neonates were more likely to develop jaundice than females. This finding is consistent with studies conducted in the Amhara region [59], Mekele [27], Malaysia [60], Nepal [19], Iran [18], and Sweden [13]. This can be attributed to the fact that some causes of neonatal jaundice are genetically linked to the X chromosome, making male babies more susceptible.

Additionally, the prevalence of glucose-6-phosphate dehydrogenase (G6PD) deficiency, which can contribute to jaundice, may be higher in males. However, further research is needed as population-based studies are scarce on G6PD deficiency, and routine G6PD screening is not commonly practiced in healthcare facilities. Moreover, males generally have higher levels of bilirubin, which can result in pathologic jaundice [7].

It is important to closely observe male neonates during the early neonatal period to enable early diagnosis and intervention for neonatal jaundice. However, it is worth noting that this finding contradicts a report from Iran [16], which could be due to differences in the study area and socio-demographic distribution. The higher representation of males in our study may also contribute to this discrepancy.

The mode of delivery was identified as another factor significantly associated with neonatal jaundice. The study revealed that cesarean delivery was found to be protective against neonatal jaundice by 76% compared to vaginal delivery. This finding is consistent with a study conducted in Iran, where vaginally delivered infants had a higher likelihood of developing newborn jaundice compared to those delivered via cesarean delivery [18]. The reason behind this association could be the possibility of birth trauma during vaginal delivery, which exposes the newborn to physical strain and increases the likelihood of bleeding and hemolysis, leading to neonatal jaundice.

Improving the follow-up of labor through the use of a partograph could potentially reduce the occurrence of neonatal jaundice. However, these findings do not align with a study conducted in Malaysia, which found a higher risk of newborn jaundice following cesarean births [60]. These variations in results could be attributed to differences in sample sizes or other unaccounted-for obstetric practices.

The study also demonstrated that premature neonates were almost four times more susceptible to jaundice compared to full-term neonates. This finding is consistent with studies conducted in India [61], America [62], Rwanda [63], and India [62]. Premature newborns have immature livers, which play a vital role in bilirubin metabolism. Their immature livers may not be able to effectively process and excrete bilirubin, resulting in its accumulation and neonatal jaundice [7]. Therefore, interventions aimed at reducing preventable causes of prematurity could be an effective approach to reducing neonatal jaundice.

Another determinant factor identified was ABO incompatibility. Neonates with ABO incompatibility were more likely to develop neonatal jaundice compared to those without. This finding is supported by a study conducted in India [62]. ABO incompatibility can lead to immune-mediated hemolysis of the newborn’s blood due to maternal antigens, which is one of the causes of infant jaundice [8]. Therefore, it is important to investigate the neonatal blood group before discharge, especially for neonates born to mothers with an O-blood group, to identify ABO incompatibility and provide appropriate advice to the mother regarding the probability of jaundice and the need to seek healthcare for the neonate.

### Limitations of the study

The first limitation of this study was it might lead to bias since it was a facility-level study. Secondly, males were highly represented in the study, which might reduce its generalizability. The study also shares the limitation of a case-control study.

## Conclusion

Governments, non-governmental organizations, health officers, and policymakers should stress mainly the management of ABO incompatibility and early detection of prematurity to prevent, diagnose, and treat neonatal jaundice. Additionally, neonates who were born with vaginal delivery and whose mothers were > 35 years old as well as those who lived in cities had a higher chance of developing newborn jaundice. Following mother-infant pairs at increased risk for neonatal jaundice, both at the time of antenatal and postnatal periods and intervening earlier is likely a key factor in decreasing severe neonatal jaundice in this study setting.

To advance the field, future researchers should address the limitations of this study by mitigating gender-related biases and enhancing our understanding of the factors linked to neonatal jaundice. Moreover, it is advisable to explore alternative study designs, as doing so can bolster the evidence, minimize biases, and provide better control over confounding variables. Additionally, it is recommended to investigate the underlying factors that contribute to the disparity in neonatal jaundice prevalence between urban and rural areas. Furthermore, researchers should delve into the influence of cultural practices and conduct comparative studies to gain insights into variations across different regions. By pursuing these avenues, we can foster a more comprehensive understanding of neonatal jaundice and its associated factors.

## Data Availability

The data sets generated and/ or analyzed in the current study are not available publicly due to the consent we took from the study participants in order not to share this data with others but are available from the corresponding author upon a reasonable request.

## Abbreviations

AOR: Adjusted Odds Ratio
APH: Antepartum Hemorrhage
CI: Confidence Interval
COR: Crude Odds Ratio
DM: Diabetes Mellitus
IMNCI: Integrated Management of Neonatal and Childhood Illness
IUGR: Intra Uterine Growth Restriction
KM: Kilo Meter
NICU: Neonatal Intensive Care Unit
PROM: Premature Rupture of Membrane
SPSS: Statistical Package for Social Science
